# The Main Anthocyanin Monomer from *Lycium ruthenicum* Murray Fruit Mediates Obesity via Modulating the Gut Microbiota and Improving the Intestinal Barrier

**DOI:** 10.3390/foods11010098

**Published:** 2021-12-30

**Authors:** Peiyun Liu, Wangting Zhou, Weiqi Xu, Yujia Peng, Yamei Yan, Lu Lu, Jia Mi, Xiaoxiong Zeng, Youlong Cao

**Affiliations:** 1 College of Food Science and Technology, Nanjing Agricultural University, Nanjing 210095, China; 2019108063@njau.edu.cn (P.L.); 2019208019@njau.edu.cn (W.Z.); 2018208024@njau.edu.cn (W.X.); 2018208023@njau.edu.cn (Y.P.); 2 Institute of Wolfberry Engineering Technology, Ningxia Academy of Agriculture and Forestry Sciences, Yinchuan 750002, China; yanyamei@163.com (Y.Y.); lulubing8901@163.com (L.L.); lorna0102@126.com (J.M.); 3 National Wolfberry Engineering Research Center, Yinchuan 750002, China

**Keywords:** *Lycium ruthenicum* Murray, anthocyanins, obesity, gut microbiota, intestinal barrier

## Abstract

Anthocyanins have been shown to exert certain antiobesity properties, but the specific relationship between anthocyanin-induced beneficial effects and the gut microbiota remains unclear. Petunidin-3-*O*-[rhamnopyranosyl-(trans-*p*-coumaroyl)]-5-*O*-(β-D-glucopyranoside) (P3G) is the main anthocyanin monomer from the fruit of *Lycium ruthenicum* Murray. Therefore, in this study, we investigated the antiobesity and remodeling effects of P3G on gut microbiota through a high-fat diet (HFD)-induced obesity mouse model and a fecal microbiota transplantation experiment. P3G was found to reduce body weight gain, fat accumulation, and liver steatosis in HFD-induced obese mice. Moreover, supplementation with P3G alleviated the HFD-induced imbalance in gut microbiota composition, and transferring the P3G-regulated gut microbiota to recipient mice provided comparable protection against obesity. This is the first time evidence is provided that P3G has an antiobesity effect by changing the intestinal microbiota. Our present data highlight a link between P3G intervention and enhancement in gut barrier integrity. This may be a promising option for obesity prevention.

## 1. Introduction

Rapidly becoming a global epidemic, obesity leads to a slew of systemic disorders, such as insulin resistance [1], type 2 diabetes [2], fatty liver disease [3] and cardiovascular disease [4], which seriously affect public health and create a huge socio-economic burden. Over 1.9 billion adults are overweight or obese [5]. As a result, the prevention and treatment of obesity have become a serious concern around the world. Generally, many risk factors result in obesity, such as genetic variance, imbalance between caloric intake and metabolic expenditure, metabolic-stress-induced systemic inflammation, and excessive oxidative stress [6]. It has been reported that a healthy gut lumen not only serves as an interface for the absorption of various nutrients, but also provides space for trillions of resident intestinal bacteria, affecting energy intake from food and immune homeostasis [7,8,9]. Hence, emerging studies have connected the variations in the gut ecosystem with diet-induced obesity.

A high-fat diet (HFD) causes aberrant alterations in gut microbial composition, leading to gut microbiota dysbiosis [10,11]. For example, Firmicutes and Bacteroidetes change in obese subjects or animals [12], characterized by an increase in the relative abundance of Firmicutes and a decrease in the relative abundance of Bacteroidetes [13,14]. Reductions in microbial richness and diversity, as well as alterations in specific bacterial families, genera, and species have also been observed in HFD-induced obesity [15,16]. These findings suggest that gut microbes may be involved in HFD-induced obesity. In addition, gut microbiota dysbiosis caused by HFD can reduce the expression of tight junction (TJ) proteins, which increases intestinal permeability and drives lipopolysaccharide (LPS) to enter the blood circulation, promoting the production of inflammatory cytokines [17] and eventually leading to systemic inflammation and insulin resistance [18].

Mounting evidence is showing that anthocyanins can reduce fat accumulation and lead to the loss of body weight by inhibiting lipid absorption [19], increasing energy expenditure, suppressing food intake, regulating lipid metabolism [20], reducing obesity-associated oxidative stress and inflammation, and so on [21,22]. As most anthocyanins cannot be digested and absorbed in the upper gastrointestinal tract, but can be utilized and converted into metabolites by gut microbiota in the large intestine, manipulating the gut microbiota by anthocyanins has thus been received considerable interest [23]. Consistent with this idea, anthocyanins have been shown to modify the composition of gut microbiota, enhancing intestinal barrier integrity and decreasing systemic inflammation, ultimately preventing the development of obesity [24,25]. *Lycium ruthenicum* Murray, a rare long-lived perennial shrub native to China’s northwest, is used in traditional medicine and cuisine [26]. *L. ruthenicum* is rich in anthocyanins (ACN), which have a variety of biological functions, showing antioxidation, anti-inflammation, and antihepatic steatosis activities [27,28,29]. In a previous study, we found that the long-term intake of ACN could regulate the gut microbiota in normal chow-diet-fed mice, accompanied by improved antioxidant status in the liver and anti-inflammatory status in the colon [30]. In addition, both ACN and its main component, petunidin-3-*O*-[rhamnopyranosyl-(trans-*p*-coumaroyl)]-5-*O*-(β-D-glucopyranoside) (P3G), reduced colitis in mice induced by dextran sodium sulfate through regulating the gut microbiota and enhancing the expression of TJ proteins [31]. In the current study, we investigated the protective impact of P3G on HFD-induced obesity in mice, and used a fecal microbial transplantation experiment to see if this positive effect was connected to the gut microbiota. The function of P3G in the preservation of intestinal barrier integrity was examined in vivo and in vitro to further understand the potential mechanisms of action of P3G.

## 2. Materials and Methods

### 2.1. Preparation of P3G

P3G was prepared according to the reported method [31]. Briefly, the dried fruits of *L. ruthenicum* were ground and extracted 3 times with 80% aqueous ethanol solution (*v/v*) for 3 h at 50 °C. The resulting solutions were filtered, concentrated, and loaded onto an AB-8 macroporous resin column (5 × 30 cm). The column was washed with deionized water to remove impurities, followed by elution with 80% aqueous ethanol solution (containing 0.1% formic acid), and the eluent was collected, concentrated, and lyophilized to afford the crude ACN. Finally, high-speed countercurrent chromatography using a two-phase solvent system of n-butanol/methyl tert-butyl ether/acetonitrile/water/trifluoroacetic acid (2:2:1:5:0.01, *v/v*) was used to purify crude ACN, producing P3G.

### 2.2. Animal Experiment

The Animal Ethics Committee of Nanjing Agricultural University accepted all present animal procedures (NJAU.No20210309010). Male C57BL/6J mice (5 weeks old), purchased from Shanghai SLAC Laboratory Animal Co., Ltd. (Shanghai, China), were kept in a controlled environment (12 h light/dark cycle, 20–25 °C, and 60% ± 5% relative humidity) with free access to food and water.

#### 2.2.1. Animal Protocol 1

At the age of 6 weeks, three groups of mice were formed at random (*n* = 6–8 each): (1) normal control (NC) group fed a normal diet (10% of energy from fat, Xie Tong Corp., Nanjing, China); (2) HFD group fed a high-fat diet (60% of energy from fat, Research Diet, New Brunswick, NJ, USA); (3) P3G group fed a HFD and oral administration of P3G (100 mg/kg body weight). The NC and HFD groups were simultaneously given sterile water by gavage daily. On the 11th week, the fasting blood glucose and oral glucose tolerance test (OGTT) were performed. At the end of the experiment, all the mice were starved for 12 h before being slaughtered. The plasma and tissues including the fat, liver, and colon were collected for biochemical analysis.

#### 2.2.2. Animal Protocol 2

For fecal microbiota transplantation, the performed procedure was as follows: 200 mg of fresh feces from HFD group or P3G group was collected every day and homogenized with 4 mL saline containing 0.5 g/L cysteine. Then, the insoluble particles were removed by centrifugation at 1000 rpm for 1 min, and the resulting supernatant was used as the transplant material. For 12 weeks, six-week-old recipient mice (*n* = 6–8) were given a HFD and transplanted once daily with the donor group’s transplant material.

### 2.3. Oral Glucose Tolerance Test (OGTT)

The mice were fasted for 8 h before receiving an intragastric gavage of a glucose solution (1.5 g/kg body weight). After gavage, the levels of blood glucose from the tail vein blood were measured using a glucometer (Roche Diagnostics, Basel, Switzerland) at the prescribed time points (0, 15, 30, 60, 90, and 120 min). To quantify the OGTT value, the area under the curve (AUC) was determined using trapezoidal methods.

### 2.4. Measurement of Colon Length and Body and Fat Weights

The body weight of each mouse was measured every week. In addition, the length of the colon and the weight of fat including subcutaneous fat, perirenal fat, epididymal fat, and mesenteric fat were measured after sacrifice.

### 2.5. Plasma Measurement

Commercial kits (Nanjing Institute of Bioengineering, Nanjing, China) were used to determine the levers of alanine transaminase (ALT), aspartate transaminase (AST), triglyceride (TG), high-density lipoprotein cholesterol (HDL-C), low-density lipoprotein cholesterol (LDL-C) and LPS in plasma. The plasma glucagon-like peptide-1 (GLP-1) level was quantified using an enzyme-linked immunosorbent assay (ELISA) kit (Meimian Biotech Co., Ltd., Yancheng, China). An ELISA kit (Mercodia AB, Uppsala, Sweden) was used to test insulin lever. ELISA kits (Nanjing Jiancheng Bioengineering Institute, Nanjing, China) were used to quantify the plasma concentrations of pro-inflammatory cytokines (interleukin (IL)-6 and IL-1β).

### 2.6. Histological Analysis

The colon and adipose tissues were fixed in 4% paraformaldehyde, cut into 5 μm thick slices that embedded in paraffin and then stained with hematoxylin and eosin (H&E). To observe lipid droplets in liver, frozen liver samples were sectioned to 8 μm thickness and subjected to Oil Red O staining. The stained samples were observed with a Panoramic MIDI scanner (Sysmex Europe GmbH, Norderstedt, Germany).

### 2.7. RNA Extraction and Quantitative Real-Time Polymerase Chain Reaction

A Tissue RNA Isolation Kit (Vazyme Biotech Co., Ltd., Nanjing, China) was used to extract total RNA according to the manufacturer’s instructions. After quantification using a NanoDrop system (Thermo Fisher Scientific, Waltham, MA, USA), the purified RNA was used as template to generate complementary DNA with a High-Capacity cDNA Reverse-Transcription Kit (TaKaRa Bio. Inc., Nojihigashi, Kusatsu, Japan). On a QuantStudio 6 real-time PCR System, SYBR Green Master Mix (Vazyme Biotech Co., Ltd., Nanjing, China) was used to a perform quantitative real-time polymerase chain reaction (qRT-PCR). The relative mRNA expression level of the target gene was measured using the 2^−ΔΔCt^ method and the housekeeping gene glyceraldehyde-3-phosphate dehydrogenase (GAPDH). The specific target genes and primer sequences are shown in Table S1.

### 2.8. Gut Microbiota Analysis

A QIAamp DNA Stool Mini Kit (QIAGEN, Hilden, Germany) was used to extract genomic DNA from feces using according to the manufacturer’s instructions. To construct an amplicon sequencing library, the V3–V4 region of the bacteria’s 16S rRNA was amplified by PCR using general bacterial primers. After purification, homogenization, and mixing, the amplicon was sequenced by 2 × 250 bp paired-end sequencing on a MiSeq platform by Genesky Biotechnologies Inc. (Shanghai, China). Raw files were subjected to QIIME 2.0 to remove barcodes, primers, and low-quality sequences. Amplicon sequence variants (ASVs) were clustered with a 97% similarity cutoff and taxonomically assigned using the Greengenes database. Alpha diversity (Observed_species, Chao1, ACE, Shannon, and Simpson indexes) was calculated by Mothur (version 1.30.1, http://.mothur.org/, 30 October 2021) to evaluate community richness [32]. Principal coordinates analysis (PCoA) of the Bray–Curtis dissimilarities was performed using R packages (version 4.1.1, http://www.r-project.org/, 30 October 2021) to visualize the compositional differences between the microbial communities of different groups. In addition, the linear discriminant analysis (LDA) effect size (LEfSe) was used to identify bacterial taxa with significant differences between groups. Furthermore, Phylogenetic Investigation of Communities by Reconstruction of Unobserved States (PICRUSt2) was applied to predict the functional features of the microbial communities [33].

### 2.9. Cell Culture

In an incubator at 37 °C and 5% CO_2_, Caco-2 cells were cultured in Dulbecco’s modified Eagle medium (DMEM) containing 10% fetal bovine serum, streptomycin (100 mg/mL), and penicillin (100 unit/mL). The cells were seeded at a density of 1 × 10^4^ cells per well in 96-well plates and incubated for 24 h. Then, the adherent cells were washed twice with phosphate buffered saline (PBS) and incubated with P3G at various concentrations (800, 400, 200, 100, 50, and 25 µg/mL) for 24 h. The cell viability was evaluated by a 3-(4,5-dimethylthiazol-2-yl)-2,5-diphenyltetrazolium bromide (MTT) assay.

### 2.10. Gene Expression

Caco-2 cells were cultured on 12-well plates for 21 days with the medium being changed every other day. At day 22, Caco-2 cells were exposed to 100 µg/mL LPS simultaneously with P3G (100, 200, 400, or 600 µg/mL) for 24 h. After the treatment, the relative mRNA expression levels of zonula occludens (ZO)-1, claudin-1, claudin-4, and Occludin were determined using qPCR. The specific target genes and primer sequences are shown in Table S2.

### 2.11. In Vitro Permeability Study

Caco-2 cells were seeded in 12-well Transwell^®^ plates (Corning, NY, USA) to grow and differentiate to form an epithelial monolayer. After 21 days, the Caco-2 monolayer cells growing in the apical chamber were treated with LPS (100 µg/mL) and P3G (100 µg/mL) for 24 h. Subsequently, the monolayer cells were then rinsed with Hank’s balanced salts solution (HBSS), and 0.5 mL of fluorescein isothiocyanate (FITC)-dextran (4 kDa, Sigma Aldrich, Saint Louis, MO, USA) solution (1 mg/mL in HBSS) was added to the apical chamber, whereas 1.5 mL HBSS was added to the basal chamber. After 6 h, the sample was collected from the basal chamber and the fluorescence intensity (excitation, 490 nm; emission, 520 nm) was measured with a multi-mode microplate reader (Thermo Fisher Scientific, Waltham, MA, USA).

### 2.12. Statistical Analysis

The experimental results are expressed as mean ± standard error (mean ± SEM). The two-tailed Student’s *t*-test was used to examine the difference between two groups. To investigate the difference between more than two groups, one-way analysis of variance (ANOVA) was used, followed by Duncan’s post hoc test. A *p*-value < 0.05 was deemed significant.

## 3. Results

### 3.1. P3G Supplementation Reduced Weight Gain and Fat Accumulation in HFD-Fed Mice

**Figure 1:**
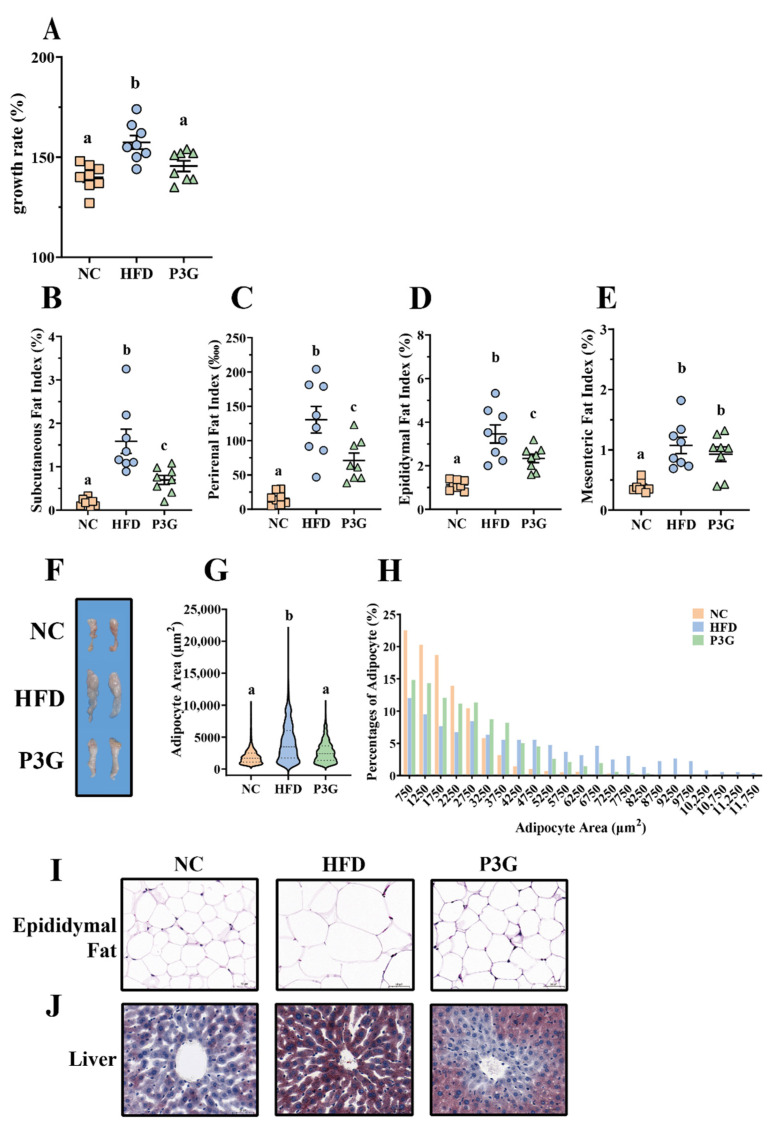
P3G supplementation reduced weight gain and fat accumulation in HFD-induced mice. Mice induced by high-fat diet were treated with P3G (100 mg/day/body weight) for 12 weeks (n = 6–8 in each group): (**A**) growth rate within 12 weeks; (**B**) subcutaneous fat index; (**C**) perirenal fat index; (**D**) epididymal fat index; (**E**) mesenteric fat index; (**F**) adipose tissue; (**G**,**H**) epididymal adipocyte size (adipocyte size was estimated using ImageJ software); (**I**) hematoxylin and eosin (H&E)-stained epididymal adipose tissue (scale bar, 100 μm); (**J**) representative pictures of Oil Red O-stained liver tissue (scale bar, 100 μm). Data are expressed as mean ± standard error (mean ± SEM) and analyzed using one-way analysis of variance (ANOVA); a, b, and c represent significant differences between groups. The same letter indicates no significance between the two groups, and different letters indicate significance (*p* < 0.05).

To observe the modulating effect of P3G on obesity, P3G was orally administered to C57BL/6J mice fed a HFD. The scheme of this experiment is shown in Figure S1A. After 12 weeks of intervention, the body weight gain of the P3G group was much lower than that of the HFD group (Figure 1A). The changes in the weight of adipose tissue were also investigated, since a clear link exists between weight gain and fat accumulation. The results showed that P3G supplementation effectively decreased the increased fat mass (including subcutaneous fat, perirenal fat, and epididymal fat) caused by the HFD (Figure 1B–F). Adipocyte size and number decreased in the epididymal white fat of the P3G group compared to those in the HFD group (Figure 1G–I), which is consistent with lower adiposity. P3G reduced the lipid deposition in liver of mice fed a HFD to a certain extent (Figure 1J), suggesting a beneficial effect of P3G on liver steatosis. In addition, P3G alleviated the reduction in colon length in HFD mice (Figure S1B,C). Collectively, these data implied that intervention with P3G could efficiently reduce the weight gain and fat accumulation induced by the HFD.

### 3.2. P3G Supplementation Improved Dyslipidemia and Glucose Homeostasis in HFD-Fed Mice

**Figure 2:**
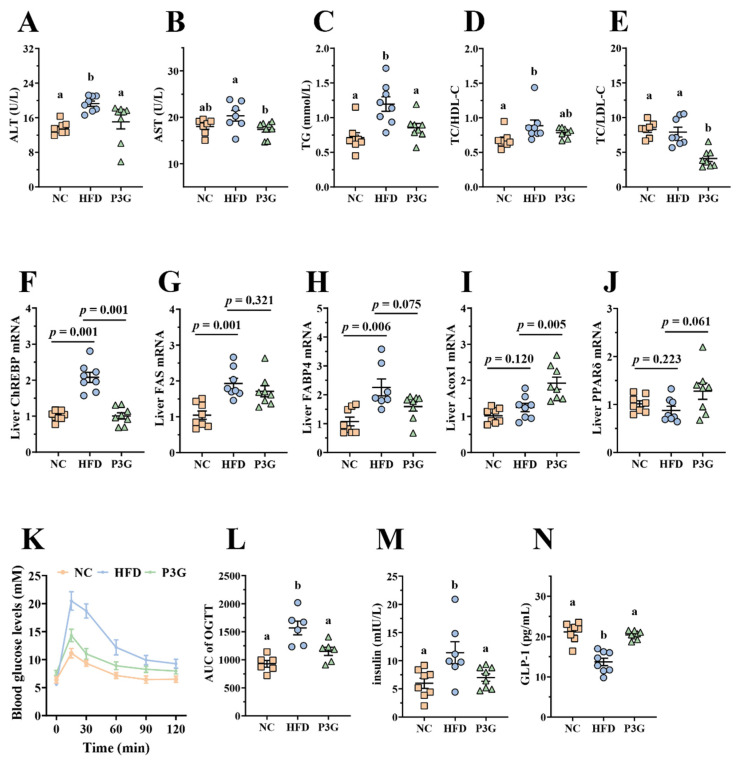
P3G supplementation alleviated HFD-induced glycolipids metabolism disruption in mice. (**A**) ALT, (**B**) AST, (**C**) TG, (**D**) TC/HDL-C, and (**E**) TC/DLD-C levels in plasma. (**F**–**J**) ChREBP, FAS, FABP4, Acox1, and PPARδ mRNA levels in liver, respectively. (**K**) Blood glucose and (**L**) area under the curve (AUC). (**M**) Plasma insulin level. (**N**) Plasma GLP-1. Data are expressed as mean ± standard error (mean ± SEM) and analyzed using one-way analysis of variance (ANOVA); a and b represent significant differences between groups. The same letter indicates no significance between the two groups, and different letters indicate significance (*p* < 0.05).

Obesity is caused by a disruption in lipid and glucose metabolism. As shown in Figure 2A–E, the plasma levels of ALT, AST, TG, and LDL-C relative to total cholesterol (TC) were largely reduced in the P3G-treated mice, indicating a noticeable protective effect of P3G on plasma lipid levels. The relative mRNA expression of genes involved in lipid metabolism in the liver was also investigated. The P3G group exhibited lower expression of carbohydrate-responsive element-binding protein (ChREBP), which is involved in lipogenesis (Figure 2F). The mRNA expression levels of the downstream genes of ChREBP, including fatty acid synthase (FAS) and fatty acid-binding protein 4 (FAbp4), were all lower in the livers of P3G-treated mice compared to those of HFD-induced mice (Figure 2G,H). However, the mRNA levels of genes associated with lipolysis, proliferator-activated receptor δ (PPARδ), and acyl-coenzyme A oxidase 1 (Acox1) were raised (Figure 2I,J). Additionally, the oral administration of P3G improved glucose tolerance and decreased insulin concentration in the HFD-fed mice (Figure 2K,M), confirming the enhancement effect of P3G on insulin sensitivity. Interestingly, the level of GLP-1, an intestinal hormone that improves blood sugar [34], was increased in P3G-treated mice (Figure 2N). These results collectively demonstrated that intake of P3G may improve the glucolipid metabolism in HFD-fed mice.

### 3.3. P3G Supplementation Maintained the Integrity of the Intestinal Barrier and Relieved Inflammation in HFD-Fed Mice

**Figure 3:**
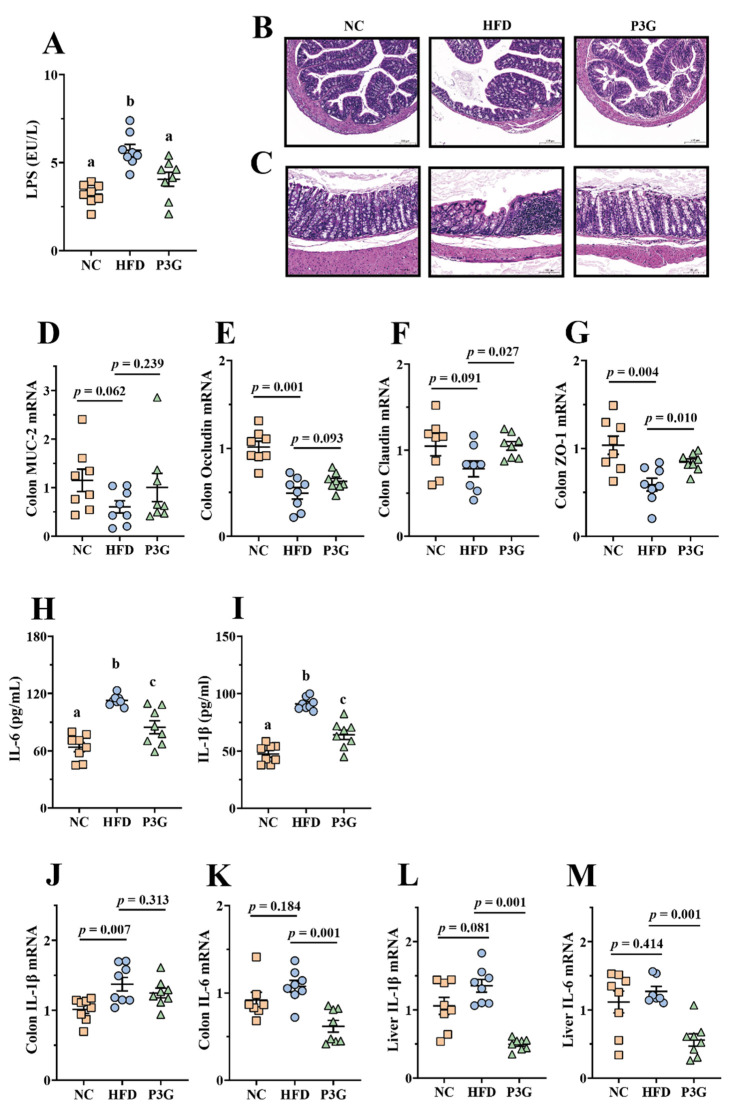
P3G relieved inflammation and improved the integrity of the intestinal barrier in HFD-induced mice: (**A**) LPS in plasma; (**B**,**C**) representative H&E pictures of colon (scale bars, 100 μm); (**D**–**G**) MUC-2, Occludin, Claudin and ZO-1 mRNA levels in the colon; (**H**,**I**) IL-6 and IL-1β in plasma; (**J**,**K**) IL-1β and IL-6 mRNA levels in the colon; (**L**,**M**) IL-1β and IL-6 mRNA levels in the liver. Data are expressed as mean ± standard error (mean ± SEM) and were analyzed using one-way analysis of variance (ANOVA); a, b, and c represent significant differences between groups. The same letter indicates no significance between the two groups, and different letters indicate significance (*p* < 0.05).

A HFD impairs intestinal barrier function and increases intestinal permeability [35], resulting in a higher level of LPS in the blood, finally triggering systemic inflammation in different organs. We found that the level of LPS in the plasma of mice induced by a HFD was considerably higher than that of the NC group, and the P3G intervention suppressed the increase in LPS (Figure 3A), showing that P3G had a protective impact on intestinal barrier integrity. Consistent with this result, the images of H&E staining of colon tissue reflected that the HFD-fed mice exhibited epithelial disruption and inflammatory cell infiltration in the submucosal layer, which were improved by P3G treatment (Figure 3B,C). Additionally, the P3G intervention significantly increased the mRNA expression levels of genes for intestinal integrity (Claudin and ZO-1) in colon tissue (Figure 3D–G). Furthermore, P3G supplementation reduced the concentrations of pro-inflammatory factors (IL-6 and IL-1β) in the plasma (Figure 3H,I), along with the suppression of the mRNA expression of IL-6 in the colon and liver (Figure 3J–M), indicating that P3G effectively alleviated barrier-dysfunction-related systemic inflammation in HFD-fed mice.

Since the intestinal barrier is thought to play a role in the antiobesity effect of P3G, the effect of P3G on the intestinal barrier was verified using LPS-induced Caco-2 cell monolayer cell permeability. We found that P3G treatment significantly upregulated the mRNA expressions of Claudin-1, Claudin-4, and Occludin in Caco-2 cells compared to those of the model control (Figure S2B–E). Furthermore, the FITC-dextran permeability assay was applied to confirm the capacity of P3G (100 μg/mL) to protect the integrity of the barrier. As expected, the results showed that the addition of P3G to the upper chamber prevented the leakage of FITC-dextran into the bottom chamber induced by LPS stimulation (Figure S2F), further demonstrating that P3G intervention can enhance gut barrier function.

### 3.4. P3G Supplementation Modified the Gut Microbial Community in HFD-Fed Mice

**Figure 4:**
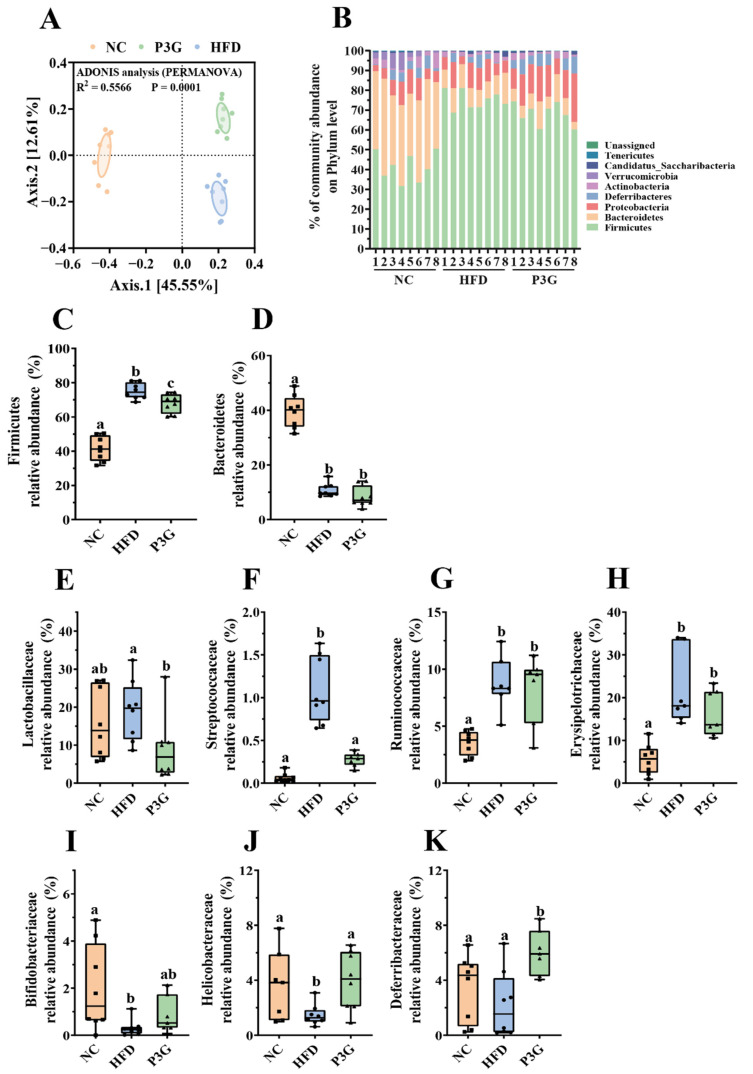
P3G supplementation regulated gut microbial community. (**A**) PCoA of gut microbiota based on the ASV data of the NC, HFD, and P3G groups. (**B**) Bacterial taxonomic profiling at the phylum level of intestinal bacteria from different groups. (**C**,**D**) The relative abundance of Firmicutes and Bacteroidetes. (**E**–**H**) Four groups of bacterial families with significantly reduced relative abundance in the P3G group compared to the HFD group. (**I**–**K**) Three groups of bacterial families with significantly increased relative abundance in the P3G group compared to the HFD group. Box-and-whisker plots are used to represent the data, and one-way analysis of variance (ANOVA) was used to evaluate the data; a, b, and c represent significant differences between groups. The same letter indicates no significance between the two groups, and different letters indicate significance (*p* < 0.05).

Changes in the gut microbiota are partially responsible for excessive LPS production and increased intestinal permeability. To determine the impact of P3G on the gut microbial community, 16S rRNA gene sequencing was used to examine the structure, abundance, and function of the gut microbiota in the feces. No significant difference in the α diversity among different groups was observed (Figure S3A–E), but the results of PCoA based on the Bray–Curtis dissimilarities indicated significant microbiota composition grouping between the NC, HFD, and P3G groups (Figure 4A), demonstrating that the HFD significantly altered the gut microbiota profiles and P3G supplementation could restore those changes.

**Figure 5:**
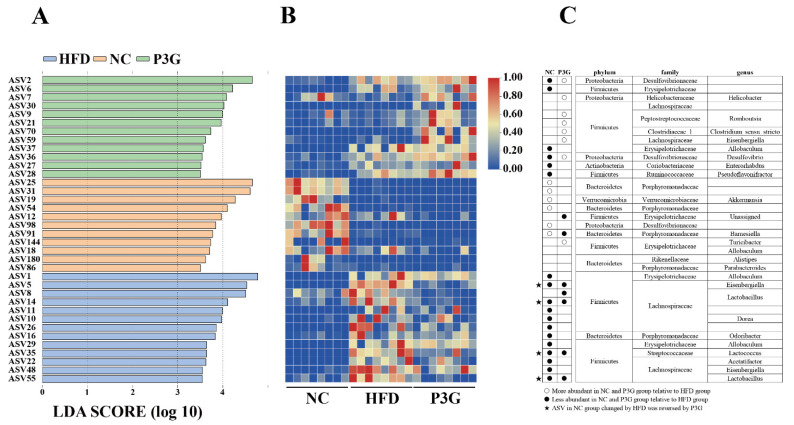
(**A**) LEfSe and (**B**) heatmap showing the most significantly different abundant taxa enriched in the microbiota from the three groups. Only the taxa with an LDA score higher than 3.5 are shown. (**C**) The circles (○) and dots (●) represent the significantly higher and lower relative abundances of ASVs in the NC or P3G group compared to the HFD group, respectively. The star (★) shows that P3G treatment restored ASV in the NC group caused by HFD intervention. The phylum, family, and genus names of ASVs are also displayed.

The dominant bacteria at the phylum level were Firmicutes, Bacteroidetes, and Proteobacteria (Figure 4B); P3G supplementation decreased the relative abundance of Firmicutes compared to the HFD group, without significant changes in the relative abundances of Bacteroidetes (Figure 4C,D). At the family level, P3G supplementation decreased the relative abundances of Lactobacillaceae, Streptococcaceae, Ruminococcaceae, and Erysipelotrichaceae in mice (Figure 4E–H). The relative abundances of Bifidobacteriaceae, Helicobacteraceae, and Deferribacteraceae in mice were increased (Figure 4I–K). Furthermore, the relative abundances of the top 20 genera were compared amongst the three groups, and we found that P3G supplementation decreased the relative abundance of *Lactobacillus* in HFD-fed mice (Figure S3F,G). Additionally, ASVs with a relative abundance of less than 0.1% in the community were excluded before LEfSe analysis, and 36 ASV-level phylotypes were identified as key variables for the largest differences amongst the three groups (Figure 5A–C). In this study, 24 ASVs differed between the HFD and P3G groups, of which 4 ASVs were adjusted by P3G, indicating that P3G can reverse microbiota dysbiosis in HFD-fed mice.

According to the results of 16S rRNA gene sequencing, the alteration of the metabolic function of the intestinal flora in mice was predicted by PICRUSt based on the Kyoto Encyclopedia of Genes and Genomes (KEGG). In the HFD group, 25 KEGG pathways were significantly upregulated compared to the NC group (Figure S4A). Notably, the metabolic pathway with significant discriminative power was bacterial chemotaxis and flagella assembly, which was higher in HFD-fed mice compared to mice fed a regular diet. P3G supplementation raised the relative abundances of 27 KEGG metabolic pathways (Figure S4B), for example, amino acid metabolism, fatty acid metabolism, sugar metabolism, vitamin metabolism, etc.

### 3.5. Gut Microbiota Contributed to the Alleviating Effects of P3G on Metabolic Disorders in HFD-Fed Mice

**Figure 6:**
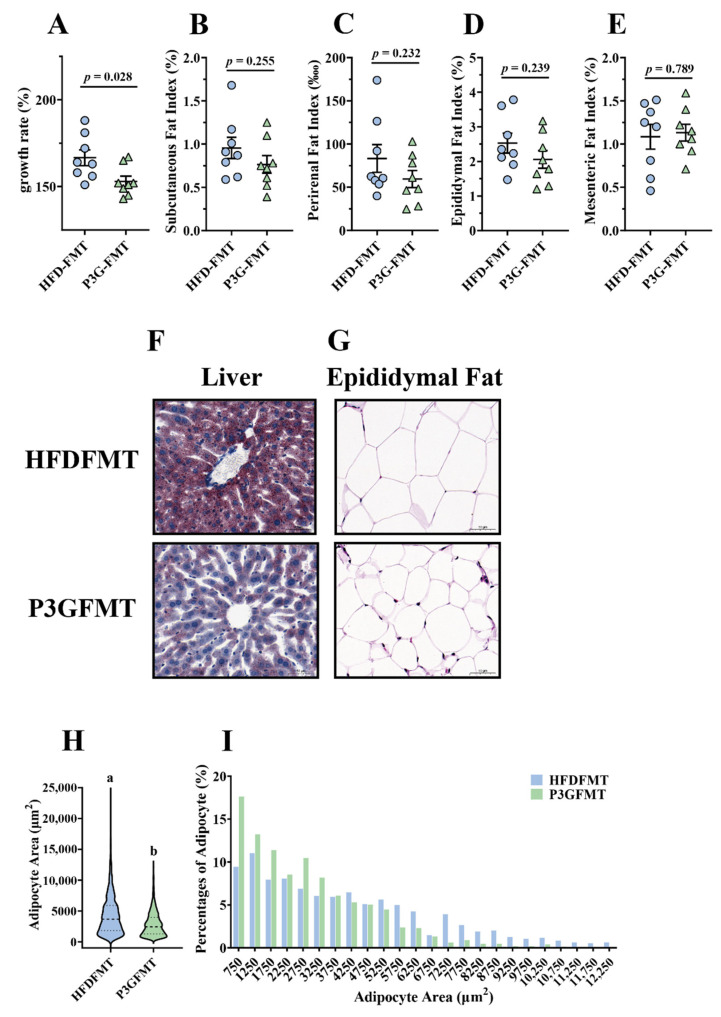
Fecal transplantation alleviated obesity and related metabolic disorders in obese mice: (**A**) growth rate within 12 weeks; (**B**) subcutaneous fat index; (**C**) perirenal fat index; (**D**) epididymal fat index; (**E**) mesenteric fat index; (**F**) representative pictures of Oil -Red-stained liver tissue (scale bar, 100 μm); (**G**) representative pictures of hematoxylin and eosin (H&E)-stained epididymal adipose tissue (scale bar, 100 μm); (**H**,**I**) epididymal adipocyte size (adipocyte size was estimated using the ImageJ software). Data are expressed as mean ± standard error (mean ± SEM) and were analyzed using the two-tailed Student’s *t*-test; a and b represent significant differences between groups. The same letter indicates no significance between the two groups, and different letters indicate significance (*p* < 0.05).

To confirm whether the P3G-induced intestinal microbial shift was involved in the improvement in obesity, a fecal microbiota transplantation (FMT) experiment was performed in HFD-fed mice for 12 weeks (Figure S5A). The body weight gain of the mice in the P3G-FMT group was lower than that of the HFD-FMT group (Figure 6A). In addition, the mice that received microbiota from P3G group exhibited lower fat content, liver fat accumulation, and fat cell size compared to the mice that received microbiota from HFD group (Figure 6B–I). Moreover, the concentration of AST in the plasma of the P3G-FMT group was lower compared to that of the HFD-FMT group, although no significant changes were observed in the levels of ALT and TG (Figure S5B–D). Additionally, mice in the P3G-FMT group showed robust improvement in glucose tolerance relative to mice in the HFD-FMT group (Figure S5E,F). Additionally, compared to the HFD-FMT group, the insulin level was lower in the P3G-FMT group, but there was no statistical difference (Figure S5G). In summary, these findings showed that the impact of P3G on HFD-induced obesity was partly mediated by the gut microbiota.

### 3.6. Fecal Transplantation Altered the Structure of Gut Microbiota in HFD-Fed Mice

**Figure 7:**
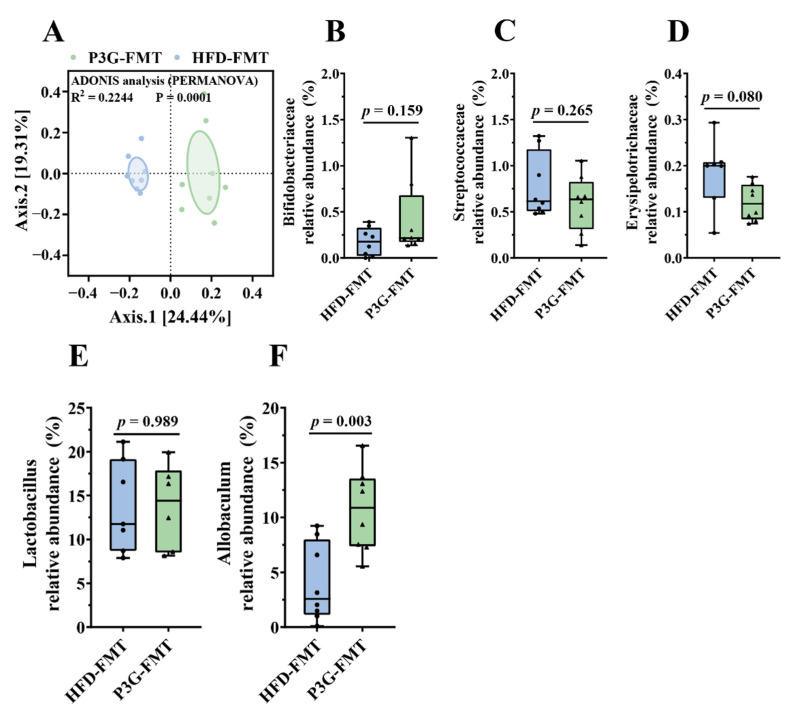

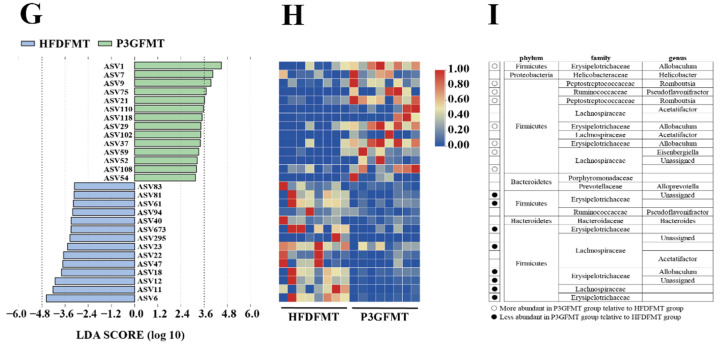
Fecal transplantation modulated the composition of intestinal microbiota. (**A**) PCoA of gut microbiota based on the ASV data of the HFDFMT and P3GFMT groups. (**B**–**D**) The relative abundance of bacterial families were different in the P3GFMT group compared to the HFDFMT group. (**E**,**F**) The relative abundance of bacterial genus were different in the P3GFMT group compared to the HFDFMT group. (**G**–**I**) LEfSe and heatmap showing the most significantly different abundant taxa enriched in the microbiota from the HFDFMT and P3GFMT groups. Only the taxa with an LDA score higher than 3.5 are shown. The circles (○) and dots (●) represent the significantly higher and lower relative abundances of ASVs in the P3GFMT group compared to the HFDFMT group, respectively. The phylum, family, and genus names of ASVs are also displayed. Box-and-whisker plots are used to represent the data, and the two-tailed Student’s *t*-test was used to evaluate the data.

The composition of the gut microbiota upon FMT was analyzed. The Simpson diversity index of the P3G-FMT group was higher than that of the HFD-FMT group (Figure S6A). Moreover, the HFD-FMT and P3GFMT groups were clearly distinguished in the PCoA (Figure 7A). Additionally, at the family level, the relative abundance of Bifidobacteriaceae was enhanced in the P3G-FMT group, and the relative abundances of Streptococcaceae and Erysipelotrichaceae were decreased (Figure 7B–D), which is consistent with the results obtained from P3G intervention in the HFD-fed mice. P3G-FMT downregulated the relative abundance of *Lactobacillus*, but upregulated the relative abundance of *Allobaculum* at the genus level (Figure 7E,F). The LEfSe results showed that 14 bacterial taxa were enriched in the HFD-FMT group, whereas 14 other bacterial taxa were increased in the P3G-FMT group (Figure 7G). Furthermore, the heatmap showed the abundances of these 28 ASVs, and simultaneously revealing that 16 ASVs were the dominant taxa responsible for these differences between two groups (Figure 7H). In general, FMT changed the gut microbiota composition in recipient mice, indicating that the P3G-mediated alteration in the gut microbiome might be involved in the mechanisms of action of P3G.

## 4. Discussion

Obesity is causing considerable concern since it is linked to metabolic disorders such as diabetes and nonalcoholic fatty liver disease [36]. Anthocyanin is a well-known natural substance that can help people lose weight and enhance human health [24,37,38]. The antiobesity effects of an anthocyanin monomer (P3G) from the fruits of *L. ruthenicum* were investigated in mice for the first time in this study. We found that administration of P3G improved lipid metabolism, glucose homeostasis, and obesity-related inflammation in mice fed a HFD. In addition, the positive effects of P3G on obesity and associated phenotypes were linked to the gut microbiota and the integrity of the intestinal barrier.

Excess dietary fat intake contributes to the development of obesity, as reflected by drastic changes in the body weight gain and physiological as well as biochemical parameters such as TC, TG, HDL-C, LDL-C, etc. [39]. One recent study confirmed that ACN supplementation lowered body weight and improved the TG and LDL-C levels in the serum of HFD-induced obesity rats [40]. Similar results were reported in mice fed a HFD [41]. However, these two studies focused on the antiobesity effects of anthocyanins mixtures. ACN contains two main anthocyanins: P3G (71.4%, relative to the total peak area in high-performance liquid chromatogram) and petunidin-3-*O*-glucoside (17.1%). The results of our present study demonstrated that P3G might partly relieve the effects obesity, as evidenced by decreased body weight (Figure 1A) and TG as well as LDL-C levels in th plasma in mice fed a HFD (Figure 2C,E). In addition, our results showed that the P3G intervention significantly reduced fat content and prevented fat cell hypertrophy (Figure 1B–E).

The liver is a crucial organ for regulating lipid metabolism and keeping the balance between lipid production and breakdown [42,43]. Studies have shown that HFD can disrupt the balance of lipid metabolism and induce the excessive accumulation of lipids in the liver, leading to hepatic steatosis [44,45,46]. P3G treatment apparently reduced lipid deposition in the livers of HFD-fed mice (Figure 1J). Simultaneously, the expressions of genes involved in lipogenesis (ChREBP and FABP4) were downregulated, and the expressions of genes involved in fatty acid oxidation (PPAPδ and Acox1) were upregulated by P3G intervention [47]. Moreover, the levels of ALT and AST in plasma, two indicators used to judge the damage degree of liver, were significantly decreased in P3G-treated mice (Figure 2A,B). These data indicated that P3G supplementation may reduce liver steatosis and restore liver function.

An impaired intestinal mucosal barrier is more prone to infection and chronic inflammation, leading to various diseases including obesity, so the integrity of the intestinal barrier is critical to host health [48,49,50]. The integrity of intestinal barrier is mediated by several TJ proteins such as ZO-1, claudin 1, and Occludin, the lack of which may increase intestinal permeability. Studies have shown that anthocyanins can regulate the intestinal TJ proteins, thereby enhancing the intestinal barrier function [25,51]. Similarly, treatment with P3G increased the mRNA expressions of ZO-1 and Claudin in the colon of HFD-fed mice (Figure 3D–G), indicating that P3G had a protective impact on the integrity of the intestinal barrier. The results of the Caco-2 cell model (Figure S2) also support this result. In addition, the increased permeability of the intestinal barrier will drive LPS to more easily enter the blood circulation, increasing proinflammatory cytokines in the plasma and different organs [52,53]. The results showed that P3G supplementation caused a significant reduction in LPS in plasma, and simultaneously reduced the levels of IL-6 and IL-1β in the plasma, colon, and liver (Figure 3H–M), suggesting that P3G supplementation improved HFD-induced inflammation.

Since the integrity of intestinal TJ can be compromised by alteration of the gut microbiota in the intestine, the interaction between P3G and the gut microbiota was further investigated. The results of PCoA showed a distinct separation between the NC, HFD, and P3G groups, indicating that both the HFD diet and P3G supplementation changed the gut microbiota composition. Firmicutes and Bacteroidetes are the two dominant phyla in the intestinal bacteria, which have different efficiencies in terms of energy extraction from foods, and play a vital role in regulating host homeostasis [54]. A HFD can cause an imbalance in the structure of the gut microbiota, as indicated by a decrease in Firmicutes abundance with an increase in Bacteroidetes abundance [55,56]. Consistent with the above reports, the findings revealed that HFD-feeding exhibited similar variations in these two phyla bacteria, whereas P3G supplementation only reduced the relative abundance of Firmicutes but had no effect on Bacteroidetes (Figure 4C,D). P3G treatment enhanced the relative abundance of Bifidobacteriaceae in the HFD group at the family level (Figure 4I), which has been reported to negatively correlate with endotoxemia [57,58]. In addition, P3G supplementation decreased the relative abundance of Streptococcaceae (Figure 4F), which has been linked to metabolic syndrome and colon cancer [59,60]. Earlier reports emphasized that the suppression of Erysipelotrichaceae could reduce HFD-induced obesity [61,62]. In agreement with those studies’ results, the relative abundance of Erysipelotrichaceae decreased in response to P3G treatment (Figure 4H), revealing that the anti-obesity effect of P3G is connected to its regulating effect on Erysipelotrichaceae. Furthermore, several species in the family Lactobacillaceae were linked to body weight reduction in HFD-induced obese mice [63], and intervention with polyphenols significantly increased the abundance of Lactobacillaceae [64,65]. However, the relative abundance of Lactobacillaceae was elevated in mice fed a HFD, while the amount of this family of bacteria was lowered in the mice treated with anthocyanins (anthocyanins from the fruits of *L. ruthenicum*) in a recent study [66]. Our findings also revealed that P3G treatment reduced the relative abundance of Lactobacillaceae, even though there was no significant difference between the NC and HFD groups (Figure 4E). We suppose that these observed differences between our study’s findings and those of the other could be attributed to diet, housing environment, and others.

Although the findings showed that P3G might modify the structure and composition of the gut microbiota in the HFD-fed mice, it remains to be determined whether the improved obesity phenotype of mice is dependent on the gut microbiota’s modulating effects. In recent years, FMT, transferring the intestinal bacteria of donor mice into recipient mice by oral gavage, has been considered a promising method for proving the cause-and-effect connection between gut microbiota and obesity [67,68]. Therefore, FMT was utilized to examine the role of P3G-induced gut microbiota in the protection against HFD-induced obesity. In the current experiment, no statistically significant difference in fat mass was observed following P3G-FMT treatment compared to HFD-FMT treatment (Figure 6B–E), whereas P3G-FMT treatment reduced hepatic lipid deposition and adipocyte size (Figure 6G–I). The oral glucose tolerance was also improved (Figure S5E,F), demonstrating that the antiobesity effect of P3G might be partly mediated by intestinal bacteria. Furthermore, the results of 16S rRNA sequencing indicated that the microbial composition of the HFD-FMT group was comparable to that of the HFD group, whereas the microbial composition of the P3G-FMT group was distinct from that of the HFD group but similar to that of the P3G group (Figure S6C). The relative abundance of Bifidobacteriaceae was increased by P3G-FMT treatment, and the relative abundances of Streptococcaceae and Erysipelotrichaceae were decreased by P3G-FMT treatment (Figure 7B–D). Moreover, P3G-FMT treatment resulted in an increase in the *Allobaculum* genus (Figure 7F), which is negatively correlated with obesity [69]. Based on those results, only a slight improvement in lipid between the two groups was found. This may be caused by the perturbation of the initial conditions of the microbiome (such as routine and sterile). Transplanting the gut microbiota to germ-free mice will provide more convincing support of this finding.

## 5. Conclusions

In summary, our present findings showed that intervention with P3G in mice fed a HFD resulted in the improvement in obesity-associated symptoms (excess fat accumulation and liver steatosis), insulin resistance, hepatic lipid metabolism (ChREBP, FAS ,and Acox1), and inflammation (IL-6 and IL-1β) compared to the mice solely fed a HFD. Moreover, this is the first time to evidence is provided that alteration in the gut microbiota plays an important role in the antiobesity effects of P3G. Furthermore, our data highlighted a link between P3G intervention and enhancement in the gut barrier integrity. Therefore, together, these results describe the potential mechanism of action of P3G against obesity based on gut microbiota regulation and gut barrier protection.

## Data Availability

The data presented in this study are available on request from the corresponding author.

## Supplementary Materials

The following supporting information can be downloaded at: https://www.mdpi.com/article/10.3390/foods11010098/s1, Figure S1: Mice induced by high-fat diet were treated with P3G (100 mg/day/body weight) for 12 weeks (n = 6–8 for each group). (A) The experimental timeline of P3G intervention. (B, C) Colon length. Data are expressed as mean ± standard error (mean ± SEM) and analyzed using one-way analysis of variance (ANOVA); a, b, and c represent significant differences between groups. The same letter indicates no significance between the two groups, and different letters indicate significance (*p* < 0.05); Figure S2: P3G protected the integrity of the intestinal barrier in vitro. (A) Cytotoxicity of P3G on Caco-2 cells. (B-E) ZO-1, Claudin-1, Claudin-4 and Occludin mRNA level in Caco-2 cell monolayers. (F) Flux of FITC-dextran in Caco-2 cells. Data are expressed as mean ± standard error (mean ± SEM) and analyzed using one-way analysis of variance (ANOVA); a, b, c and d represent significant differences between groups. The same letter indicates no significance between the two groups, and different letters indicate significance (*p* < 0.05); Figure S3: P3G supplementation regulated gut microbial community. (A) Chao1, (B) Observed species, (C) ACE, (D) Shannon and (E) Simpson are used to reflect alpha diversity. (F) Bacterial taxonomic profiling at the genus level of intestinal bacteria from different groups. (G) The relative abundance of Lactobacillus. Box-and-whisker plots are used to represent the data, and one-way analysis of variance (ANOVA) was used to evaluate the data; a, and b represent significant differences between groups. The same letter indicates no significance between the two groups, and different letters indicate significance (*p* < 0.05); Figure S4: Predicted and functions annotation based on 16S rRNA data was performed by PICRUSt and STAMP. (A) Effects of HFD treatment on predicted functions in NC-treated mice. (B) Effects of P3G treatment on predicted functions in HFD-treated mice; Figure S5: Fecal transplantation alleviated obesity and related metabolic disorders in obese mice. (A) The experimental timeline of FMT. (B) ALT, (C) AST and (D) TG in plasma. (E) Blood glucose and (F) area under curve (AUC). (G) Plasma insulin level. Data are expressed as mean ± standard error (mean ± SEM) and were analyzed using the two-tailed Student’s *t*-test; a and b represent significant differences between groups. The same letter indicates no significance between the two groups, and different letters indicate significance (*p* < 0.05); Figure S6: Fecal transplantation modulated the composition of intestinal microbiota. (A) Simpson. (B) Bacterial taxonomic profiling at the phylum level of intestinal bacteria from different groups. (C) PCoA analysis of gut microbiota based on the ASV data of NC, HFD, P3G, HFDFMT and P3GFMT groups. Box-and-whisker plots are used to represent the data, and the two-tailed Student’s *t*-test was used to evaluate the data; Table S1: Target genes and primers sequence used for animal study; Table S2: Target genes and primers sequence used for cells study; Table S3: Body weight gain (Mean ± SEM); Table S4: Body weight gain (Mean ± SEM).
