# Supplementary material for: The Main Anthocyanin Monomer from Lycium ruthenicum Murray Fruit Mediates Obesity via Modulating the Gut Microbiota and Improving the Intestinal Barrier
Source: Foods. 2021 Dec 30;11(1):98. doi: 10.3390/foods11010098 (PMC8750395; doi:10.3390/foods11010098)
Supplement: Supplementary file 1 [file foods-11-00098-s001.zip › foods-1492429-supplementary.pdf]

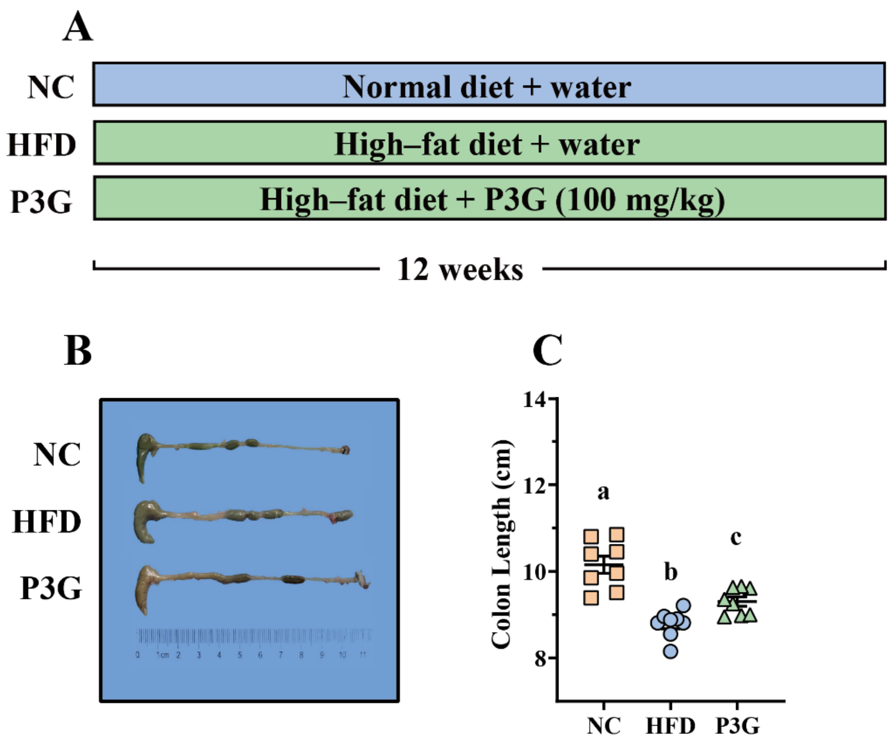

Figure S1. Mice induced by high-fat diet were treated with P3G (100 mg/day/body weight) for 12 weeks (n = 6-8 for each group). (A) The experimental timeline of P3G intervention. (B, C) Colon length. Data are expressed as mean ± standard error (mean ± SEM) and analyzed using one-way analysis of variance (ANOVA); a, b, and c represent significant differences between groups. The same letter indicates no significance between the two groups, and different letters indicate significance ($p < 0.05$).

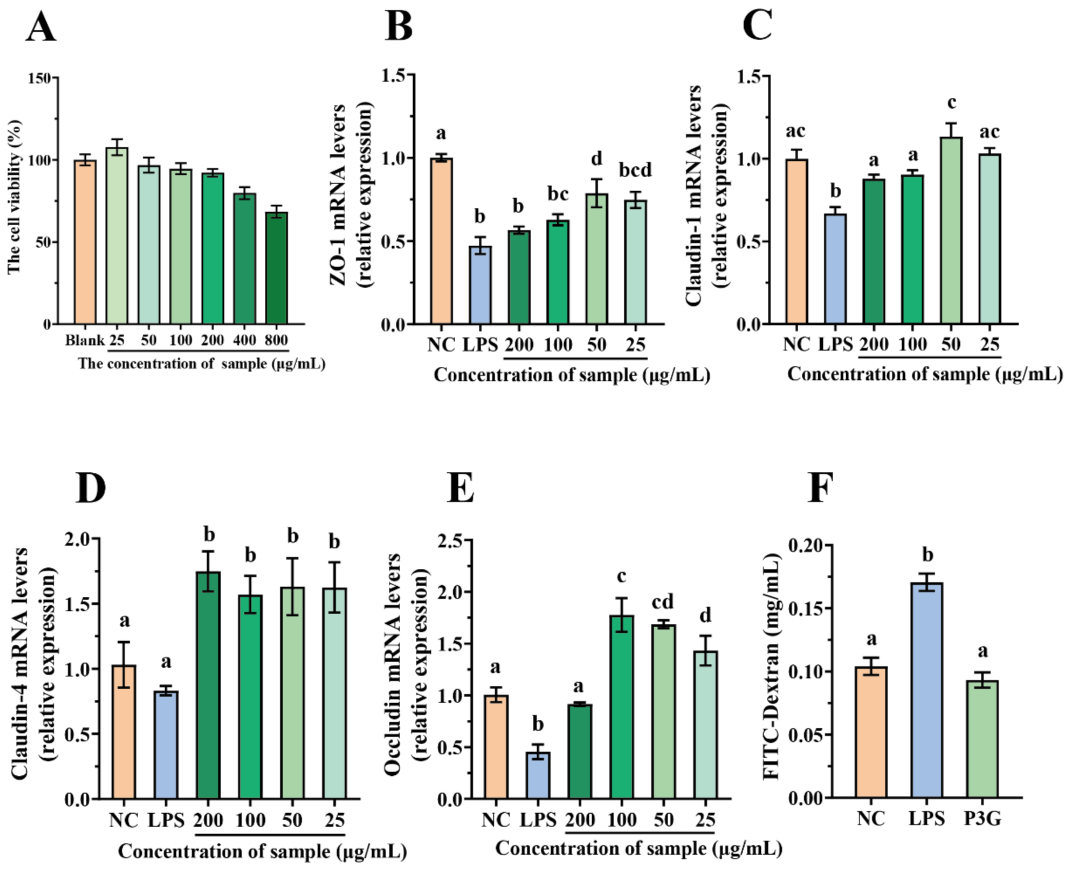

Figure S2. P3G protected the integrity of the intestinal barrier in vitro. (A) Cytotoxicity of P3G on Caco-2 cells. (B-E) ZO-1, Claudin-1, Claudin-4 and Occludin mRNA level in Caco-2 cell monolayers. (F) Flux of FITC-dextran in Caco-2 cells. Data are expressed as mean ± standard error (mean ± SEM) and analyzed using one-way analysis of variance (ANOVA); a, b, c and d represent significant differences between groups. The same letter indicates no significance between the two groups, and different letters indicate significance ($p < 0.05$).

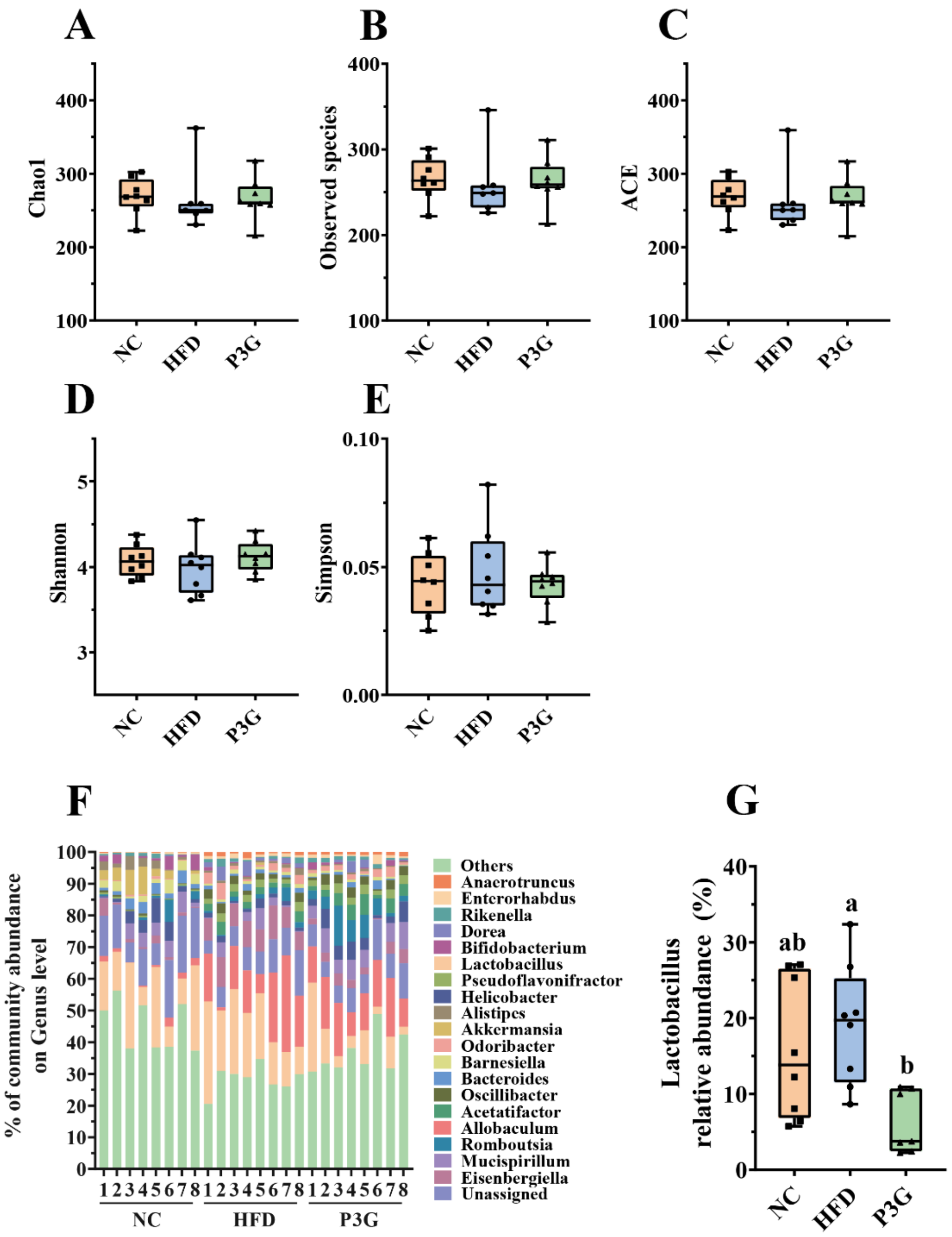

Figure S3. P3G supplementation regulated gut microbial community. (A) Chao1, (B) Observed species, (C) ACE, (D) Shannon and (E) Simpson are used to reflect alpha diversity. (F) Bacterial taxonomic profiling at the genus level of intestinal bacteria from different groups. (G) The relative abundance of Lactobacillus. Box-and-whisker plots are used to represent the data, and one-way analysis of variance (ANOVA) was used to evaluate the data; a, and b represent significant differences be-tween groups. The same letter indicates no significance between the two groups, and different letters indicate significance ($p < 0.05$).

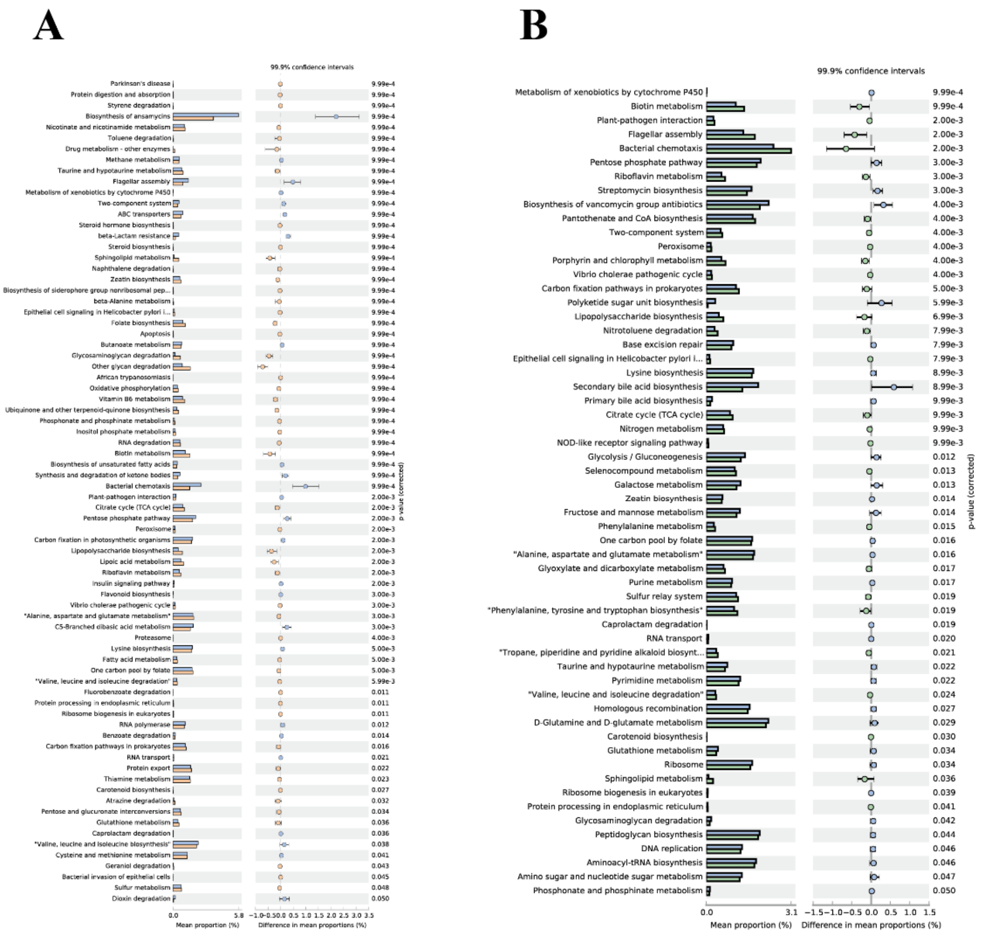

Figure S4. Predicted and functions annotation based on 16S rRNA data was performed by PICRUSt and STAMP. (A) Effects of HFD treatment on predicted functions in NC-treated mice. (B) Effects of P3G treatment on predicted functions in HFD-treated mice.

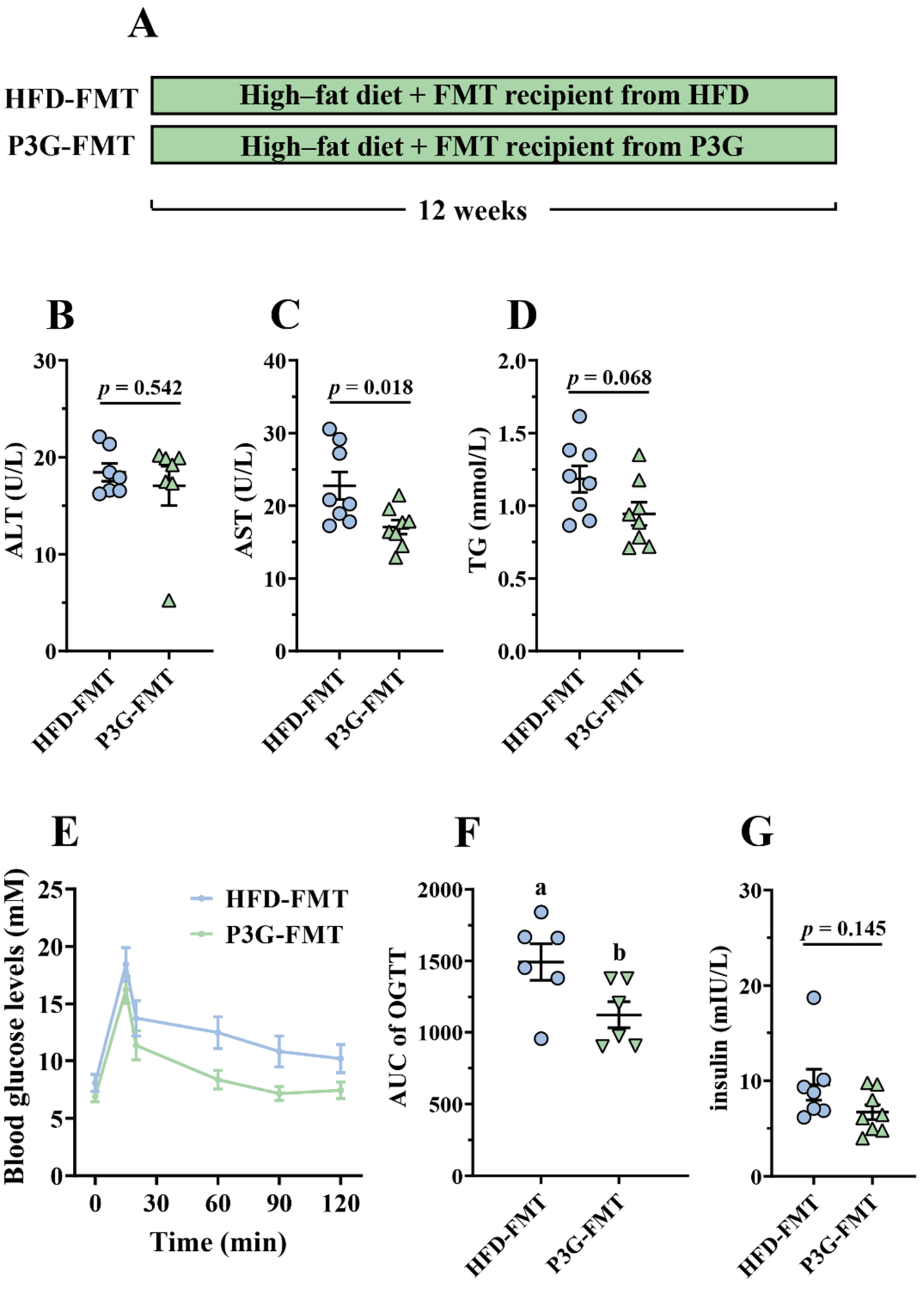

Figure S5. Fecal transplantation alleviated obesity and related metabolic disorders in obese mice. (A) The experimental timeline of FMT. (B) ALT, (C) AST and (D) TG in plasma. (E) Blood glucose and (F) area under curve (AUC). (G) Plasma insulin level. Data are expressed as mean ± standard error (mean ± SEM) and were analyzed using the two-tailed Student's t-test; a and b represent significant differences between groups. The same letter indicates no significance be-tween the two groups, and different letters indicate significance ($p < 0.05$).

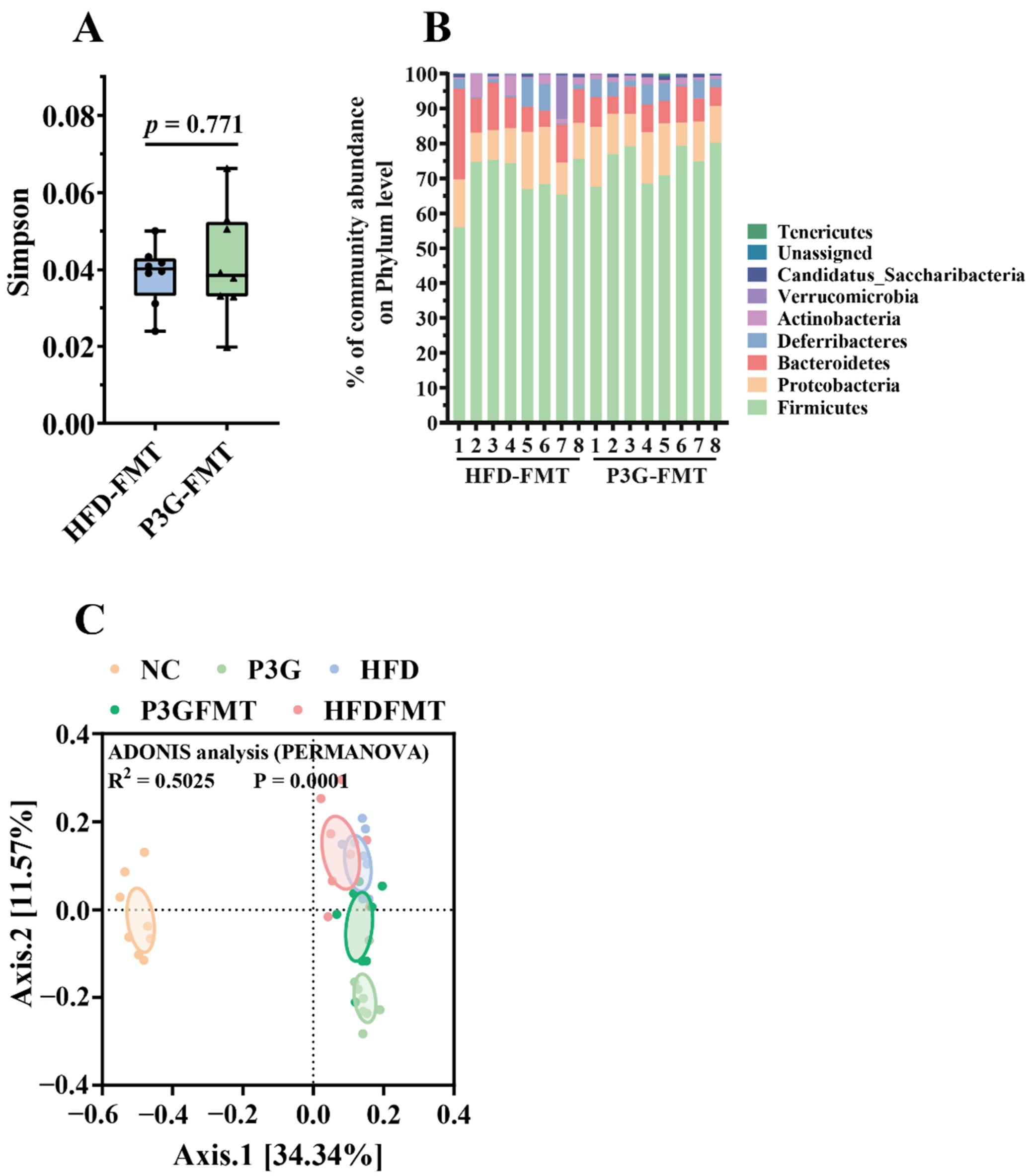

Figure S6. Fecal transplantation modulated the composition of intestinal microbiota. (A) Simpson. (B) Bacterial taxonomic profiling at the phylum level of intestinal bacteria from different groups. (C) PCoA analysis of gut microbiota based on the ASV data of NC, HFD, P3G, HFDFMT and P3GFMT groups. Box-and-whisker plots are used to represent the data, and the two-tailed Student's t-test was used to evaluate the data.

Table S1. Target genes and primers sequence used for animal study.

Target genes	Primer sequence (5'-3')
IL-1β	FW: AGCTTCAAATCTCGCAGCAG<br>RV: TCTCCACAGCCACAATGAGT
IL-6	FW: TCCATCCAGTTGCCTTCTTG<br>RV: TTCCACGATTTCCCAGAGAAC
MUC-2	FW: TGTGGCCTGTGTGGGAACTTT<br>RV: CATAGAGGGCCTGTCCTCAGG
Occludin	FW: ATGTCCGGCCGATGCTCTC<br>RV: TTTGGCTGCTCTTGGGTCTGTAT
Claudin	FW: AGCTGCCTGTTCCATGTACT<br>RV: CTCCCATTTGTCTGCTGCTC
ZO-1	FW: TTTTTGACAGGGGGAGTGG<br>RV: TGCTGCAGAGGTCAAAGTTCAAG
ChREBP	FW: TGCGGGACATGTTTGATGAC<br>RV: ATGAGGATGCTGAACACCCA
FAS	FW: GGCACCTATGGCGAGGACTT<br>RV: GCCCTCCCGTACACTCACTC
FABP4	FW: CTTTGTGGGAACCTGGAAGC<br>RV: ATGATCATGTTGGGCTTGGC
Acox1	FW: AGTCAAAGGCATCCACCAAAG<br>RV: CTATGGGATCAGCCAGAAAGG
PPAPδ	FW: TCTGCCATCTTCTGCAGCAGCTT<br>RV: CTCTTCATCGCGGCCATCATTCT
GAPDH	FW: AGGTCGGTGTGAACGGATTTG<br>RV: TGTAGACCATGTAGTTGAGGTCA

Table S2. Target genes and primers sequence used for cells study.

Target genes	Primer sequence (5’-3’)
ZO-1	FW: ATCAGGGACATTCAATAGCGTAGC
	RV: CAAGATAGTTTGGCAGCAAGAGATG
Occludin	FW: CGCTGCTGTAACGAGGCT
	RV: CCAATGTCGAGGAGT
Claudin-1	FW: AATTCGTACCTGGCATTGACTGG
	RV: TGGTGGTGGGCATCCTCCTG
Claudin-4	FW: CGTCCATCCACTCTGCACTT
	RV: TCTCCTCTGTTCCGGGTAGG
GAPDH	FW: GCGCCCAATACGACCAAATC
	RV: GACAGTCAGCCGCATCTTCT

Table S3. Body weight gain (Mean ± SEM).

	NC	HFD	P3G
**week 0**	100 ± 0 [a]%	100 ± 0 [a]%	100 ± 1[a]%
**week 1**	106 ± 2 [a]%	111 ± 2 [b]%	105 ± 2 [a]%
**week 2**	111 ± 4 [a]%	117 ± 3 [b]%	110 ± 4 [a]%
**week 3**	115 ± 4 [a]%	122 ± 3 [b]%	115 ± 4 [a]%
**week 4**	120 ± 5 [a]%	126 ± 3 [b]%	120 ± 5 [a]%
**week 5**	126 ± 6 [a]%	135 ± 3 [b]%	126 ± 6 [a]%
**week 6**	126 ± 6 [a]%	143 ± 5 [b]%	131 ± 5 [a]%
**week 7**	127 ± 6 [a]%	149 ± 6 [b]%	134 ± 6 [a]%
**week 8**	129 ± 5 [a]%	150 ± 7 [b]%	136 ± 6 [c]%
**week 9**	132 ± 6 [a]%	153 ± 6 [b]%	137 ± 6 [a]%
**week 10**	130 ± 6 [a]%	156 ± 6 [b]%	138 ± 6 [c]%
**week 11**	136 ± 6 [a]%	154 ± 8 [b]%	142 ± 7 [a]%
**week 12**	140 ± 7 [a]%	158 ± 10 [b]%	146 ± 7 [a]%

Table S4. Body weight gain (Mean ± SEM).

	HFDFMT	P3GFMT
**week 0**	100 ± 0 [a] %	100 ± 0 [a] %
**week 1**	109 ± 1 [a] %	113 ± 5 [b] %
**week 2**	113 ± 2 [a] %	118 ± 6 [a] %
**week 3**	118 ± 3 [a] %	124 ± 7 [a] %
**week 4**	123 ± 3 [a] %	129 ± 6 [b] %
**week 5**	134 ± 4 [a] %	137 ± 7 [a] %
**week 6**	144 ± 6 [a] %	139 ± 6 [a] %
**week 7**	151 ± 8 [a] %	142 ± 7 [b] %
**week 8**	155 ± 11 [a] %	147 ± 7 [a] %
**week 9**	156 ± 11 [a] %	146 ± 7 [a] %
**week 10**	159 ± 11 [a] %	149 ± 7 [a] %
**week 11**	160 ± 10 [a] %	152 ± 7 [a] %
**week 12**	167 ± 13 [a] %	153 ± 9 [b] %
